# Predictive Validity of Operationalized Criteria for the Assessment of Criminal Responsibility of Sexual Offenders With Paraphilic Disorders—A Randomized Control Trial With Mental Health and Legal Professionals

**DOI:** 10.3389/fpsyg.2020.613081

**Published:** 2020-12-18

**Authors:** Sascha Dobbrunz, Anne Daubmann, Jürgen Leo Müller, Peer Briken

**Affiliations:** ^1^Institute of Sex Research, Sexual Medicine and Forensic Psychiatry, University Medical Center Hamburg-Eppendorf, Hamburg, Germany; ^2^Institute of Medical Biometry and Epidemiology, University Medical Center Hamburg-Eppendorf, Hamburg, Germany; ^3^Professorship for Forensic Psychiatry and Psychotherapy, Georg-August-University Göttingen, Göttingen, Germany

**Keywords:** assessment, criminal responsibility, sexual violence, sexual offender, paraphilic disorders

## Abstract

The prevention of sexual violence is a major goal of sexual health. In cases of accused sexual offenders, the assessment of diminished criminal responsibility of the accused is one of the most important procedures undertaken by experts in the German legal system. This assessment follows a two-stage method assessing first the severity of a paraphilic disorder and then second criteria for or against diminished capacity. The present study examines the predictive validity of two different sets of criteria for the assessment of criminal responsibility in the context of paraphilic disorders combined with sexual offending. Two exemplary case vignettes of two suspected sexual offenders were developed to assess the criteria. For each participant, one of the two exemplary case vignettes was randomly presented. The presentation of the two different sets of criteria was also randomized, so that each participant was assigned only one of the two criteria sets to rate one of the presented cases. *N* = 349 participants from different professional backgrounds (mental health and legal professionals) completed their assessments and were included in the data analysis. The data were evaluated using logistic regression. Results show that the more recently published criteria set (Briken and Müller, 2014) predicts both the severity of the disorder as well as the diminished capacity twice as good as the older criteria set of Boetticher et al. (2005) currently used regularly for forensic court reports. In preliminary conclusion, the new criteria of Briken and Müller (2014) form an empirically based assessment of criminal responsibility. However, the proposed criteria cannot replace an extensive exploration of the accused person and a careful file analysis. Validity and reliability of the results are also limited due to the methodical choice of a vignette study.

## Introduction

The assessment of criminal responsibility is subject to the individual national legal system and is therefore handled heterogeneously. It represents, besides matters of legal prognosis of criminal offenders and of credibility assessment, the most common task for psychiatric and psychological experts in German court proceedings. In the field of predicting legal-prognostic issues in sexual offenders, actuarial-statistical methods, whose conclusions are based on the application of an explicit numerical method with empirically validated items, have proven to be superior to clinically intuitive assessments (Hanson and Morton-Bourgon, 2009). Hanson and Morton-Bourgon (2005) showed that deviant sexual interests are one of the most powerful predictors of sexual recidivism. According to Levenson (2004), forensic experts assessing sex offenders show a rather poor inter-rater-reliability (IRR) of DSM-IV-TR diagnoses. However, Packard and Levenson (2006) pointed out that the informative value of Cohen’s kappa is limited and that it tends to underestimate the agreement of the raters. More common diagnoses result in greater agreement than those diagnoses that are less common or those that are based on vague or confoundable criteria, i.e., sadism. This inconsistency in diagnoses might be the result of imprecise diagnostic criteria or an indication of the low prevalence of the disorders in question, another potential factor could be the experience of the experts.

The exceptional relevance of the agreement of diagnoses in the legal context must be taken into account given the large impact they might have on the life of the person being assessed. Therefore, Marshall (2006) suggested the agreement of the raters should be reflected in a kappa of at least *K* = 0.90 when diagnosing sexual offenders. In less severe cases, the IRR should reach at least 0.60. In multiple cases of paraphilias, this could not be confirmed by either Levinson (2004) or Marshall et al. (2002). IRR of 15 forensic psychiatrists concerning the diagnosis of sexual sadism was very poor with a coefficient of *K* = 0.14. Accordingly, Marshall (2006) concluded that DSM-IV-TR diagnoses cannot be considered helpful in defining an appropriate treatment or prognosis for sexual offenders.

According to §20 of the German penal code (*Strafgesetzbuch; StGB)*, which has the status of a uniform federal law, someone who “acts without guilt [who,] when committing the act due to a pathological mental disorder, due to a profound consciousness disorder, due to intellectual deficiency or other serious mental abnormality (so called SASA), unable to see the wrongdoing or to act on this insight” should receive appropriate legal treatment: “If the offender’s ability to perceive the wrongdoing or act on this basis is significantly reduced for one of the reasons specified in §20 when the offense is committed, the sentence may be reduced” (§21 of the German penal code).

Culpability is not explicitly named in German legal text. Instead, the legislator provides mental conditions or situations which could indicate a reduced culpability. These include the inability to perceive wrongdoing or to act on this basis due to:

– A pathological mental disorder,
– A profound consciousness disorder,
– An intellectual deficiency or
– A serious mental abnormality (SASA).

The assessment of criminal responsibility takes place in a so-called two-stage method (Jescheck and Weigend, 1996). First, there is a possible assignment to one of the four entry criteria: pathological mental disorder, profound consciousness disorder, intellectual deficiency, or other severe mental abnormality (SASA). In the second step, the effects of the aforementioned conditions on the ability to understand and control (diminished capacity) are considered. The impact on the life of the person being examined as a result of the assessment can be vast. One potential consequence is the indefinite placement in a psychiatric clinic in accordance to § 63 StGB.

The assessment of the respective symptoms to the SASA and the ability to understand and control (diminished capacity) is unfortunately subject to a relatively subjective assessment. Paraphilic disorders are represented by the criterion of the SASA and hence might lead to a potentially significant reduction in the ability to control one’s own behavior (diminished capacity) which can result in diminished capacity within the meaning of §21 StGB (Schreiber and Rosenau, 2015). Ultimately, the court determines whether someone should be held legally responsible for the accused crime. The court can be advised by an external psychiatric or psychological expert on questions of these issues.

Sexual delinquency does of course not only occur as a symptom of paraphilic disorders. Other mental disorders that might lead to such behavior include, for example, intellectual developmental disorders or schizophrenia spectrum disorders.

The prevalence rate of paraphilic disorders displays distinct differences depending on the sample. A study of an Austrian population of male incarcerated sex offenders with a sample of 1346 is particularly worth mentioning here (Eher et al., 2019). The study found that 43.3% of the investigated men met the criteria for any kind of paraphilic disorder, 4.4% had a sexual sadistic disorder, 34.5% had a pedophilic disorder, and 2.8% had an exhibitionistic disorder. Regarding the general public however, the following lifetime prevalence for at least once occurring paraphilic behavior (not to be confused with the diagnosis of a paraphilic disorder) was found: 25.0% for some kind of paraphilic behavior, 2.7% for sadistic behavior, 0.9% for paraphilic behavior, and 4.3,% for exhibitionistic behavior (Baur et al., 2016).

Traditional evaluation criteria regarding the entry criteria of the SASA refer to a psychologically heterogeneous clinical presentation, and refer mainly to personality disorders without explicit reference to paraphilic disorders (Saß, 1985; Rasch, 1999; Schmidt, 2008).

The minimum requirements for the assessment of criminal responsibility (Boetticher et al., 2005) were created by an interdisciplinary group of experts. They are based on criteria derived from various theoretical models from a multitude of heterogeneous sources (Saß, 1985), and on the effects of personality pathologies on the ability to control one’s behavior. Despite these criteria only being named and not further operationalized, they do illustrate psychological states and their effects. The following criteria are indicators for a severe paraphilic disorder (Boetticher et al., 2005; Briken et al., 2019):

– The paraphilic disorder determines the sexuality of the person to a great extent, or
– Paraphilic impulses are denied and perceived as egodystonic (=alien). While being overcontrolled and masked the vast amount of time, the ability to control the impulses decreases significantly under certain circumstances.
– The paraphilic disorder is progressive with regard to its dynamic, that is, the person being evaluated experiences themselves as progressively being flooded by stronger and stronger paraphilic impulses: The pressure to act on them increases.
– The person has (as a result of particular personality factors or sexual dysfunction) few or no other opportunities to satisfy himself or herself sexually.

Indicators for and against a forensically relevant capacity to control one’s behavior in relation to paraphilic disorders in the case of sex offenses (Boetticher et al., 2005; Briken et al., 2019) are:

Indicators pointing toward a considerable level of impairment in the capacity to control one’s behavior:

– Conflictual escalation and emotional weakening before the suspected offense with an already long-standing hopelessness in the dynamic of sexual drive.
– Carrying out of the offense, even in social situations that are strictly monitored.
– An abrupt, impulsive course of criminal action.
– Criminal action that appears ritualized. Outer stimuli appear to be blanked out.
– Contributing factors (substance intoxication, co-occurring disorders, personality disorders, limited intelligence).

Indicators pointing away from a considerable level of impairment in the capacity to control one’s behavior:

– Indicators for preparations being made for the offense in advance, as well as a planned approach (elaborations of paraphilic fantasies are expressly not included here).
– The ability to wait or a crime that is very prolonged in nature.
– The crime involves a complex step-by-step sequence of actions.
– Precautions are taken against being discovered.
– The person has previously acted differently in comparable situations.

Initially, Briken and Müller (2014) suggested criteria that could reflect the severity of the paraphilia and the extent of accountability. These criteria were already standardized due to their application in prognosis instruments, and thus could possibly improve the IRR when assessing SASA and the capacity to control one’s behavior. The authors chose eight items from two established standardized prognosis instruments for sex offenders, which are operationalized in detail: from the STABLE-2007 (Matthes and Rettenberger, 2008b) and from the ACUTE-2007 (Matthes and Rettenberger, 2008a).

The following criteria are indicators to determine the severity of a paraphilic disorder:

– Paraphilic sexual interests,
– Sex drive/sex preoccupation,
– Sex as coping,
– Capacity for stable relationships,
– General social rejection.

Indicators regarding a forensically relevant capacity to control one’s behavior in relation to paraphilic disorders in the case of sex offenses are:

– Collapse of social support,
– Emotional collapse,
– Sexual pre-occupations.

Briken and Müller (2014) criteria were examined and elaborated in a further step by Brunner et al. (2016) with regard to their applicability and IRR. This pilot study showed that the criteria can be applied. However, besides a small sample size, the missing inclusion of expertise from the legal side emphasizes the need for future research. Therefore, Dobbrunz et al. (2020) examined the IRR of the two criteria catalogs in a preliminary study. Fourteen experts from the fields of psychology, psychiatry, and prosecution, who were qualified for the assessment of criminal responsibility, participated in the study. Dobbrunz et al. (2020) found that the IRR was higher based on Briken and Müller (2014) criteria than on those of Boetticher et al. (2005). It was shown that the subjective importance/relevance of the two assessment scales’ 22 criteria were rated, on average, with at least moderate accordance between the experts.

## Current Study

The aim of this work is to compare the existing operationalized criteria (Boetticher et al., 2005; Briken and Müller, 2014) for assessing the criminal responsibility of accused offenders with paraphilic disorders, which are used by various professional groups (psychologists, psychiatrists, and prosecutors) dealing with the subject of assessment of criminal responsibility. For this experimental study, we focus exclusively on the influence of paraphilic disorders, for which in the German legal system the question of criminal responsibility is much less clear than for other disorders, for example, the presence of an acute schizophrenic disorder. In order to keep the methodological design as clear and unambiguous as possible, the complexity was also reduced and other constellative factors such as intoxication were omitted in the case vignettes and in the evaluation criteria. Of course, this makes the cases less naturalistic. The criteria are examined with regard to their predictive validity in order to increase the quality and transparency of assessments, and thus achieving a substantial added value for the assessment of criminal responsibility in practice. In this study, the predictive validity based on the criteria of Boetticher et al. (2005) and Briken and Müller (2014), regarding the assessment of criminal responsibility (SASA and diminished capacity), is examined.

## Materials and Methods

For the assessment of the criminal responsibility criteria, two exemplary case descriptions of two alleged sex offenders were developed. Both case descriptions were constructed by the authors. Each case was constructed with a specific aim: the first case was constructed in a way that it would indicate no SASA and no diminished capacity, and the second was constructed so that it would indicate SASA and diminished capacity. It was important not to construct the cases in such a way that this intent was too obvious, but present cases as realistic as possible with the limitations mentioned above (no additional relevant psychiatric disorders or constellative factors). This had been assessed by the experts in the preliminary study as well (Dobbrunz et al., 2020). Thus, the decision of the experts from the preliminary study (Dobbrunz et al., 2020) that used the same case vignettes was the standard (correct assessment) regarding the SASA and the diminished capacity presented in both case vignettes. The case vignettes are shown in the Appendix.

For the verification of predictive validity, psychiatrists, clinical psychologists, and legal psychologists as well as judges and prosecutors from all over Germany were contacted by email and asked to participate in the study. These are professional groups that potentially have to deal with questions regarding the assessment of culpability in the course of their professional activities either as experts or in the context of legal decisions. We have deliberately not tried to concentrate only on participants already experienced in the assessment of criminal responsibility. Participants were recruited via relevant email distribution lists from the professional associations of the respective occupational groups. In the case of judges and prosecutors, the ministries of justice of the 16 federal states were asked for permission for the prosecutors to participate, of whom 10 federal states agreed to support the study.

An online study using LimeSurvey was carried out. LimeSurvey is a free online survey application. For each participant, one of the two exemplary case vignettes was randomly presented to assess criminal responsibility. Since no experience in assessing or working with individuals with paraphilic disorders was a prerequisite for participation in the study, the ICD-10 criteria for paraphilic disorders were also shown and were available when participants rated their assessment. The presentation of the criteria by Boetticher et al. (2005) or the criteria of Briken and Müller (2014) was also randomized, so that each participant used and evaluated only one of the two scales. The 22 items were assessed on three-level rating scales (version: is not present at all/is somewhat present/is completely present) and with regard to the assessment of the SASA as well as in the case of diminished capacity in the form of a dichotomous version (versions: exists/does not exists). Finally, in form of an open response format, the raters were asked to provide additional information on the evaluation of the individual items or to indicate which other criteria could be considered. Power and sample size calculation was carried out using the statistical software *PASS* (version: 15.03). The desired minimum sample size was *N* = 300. In total, there were four experimental groups (two case vignettes × two criteria catalogs). Logistic regression models were calculated to determine the predictive validity, using the statistical software SPSS (version 26).

### Participants

A total of 718 participants took part in the online study, 360 of whom canceled the study or did not complete the study. Another 358 participants completed the study. Of these 358 participants, nine cases were excluded because these participants were part of occupational groups that did not belong to the target groups, or because their occupational status was “student.” The sample size is therefore *n* = 349. There were 202 women (57.9%), 146 men (41.8%), and one Trans-/Inter person (0.3%). The distribution of the occupational groups participated in the investigation was: 155 psychologists (44.4%), 68 psychiatrists (19.5%), 114 judges or public prosecutors (32.7%), and 12 others (six criminologists, four scientific officers not otherwise specified, and two experts not otherwise specified; 3.4%).

The average age of the participants was M = 45.82 (SD = 11.61), with a minimum of 24 and a maximum of 77 years. The distribution of the four groups which were randomly assigned was: case vignette “rape” and criteria according to Boetticher et al. (2005): 89 participants; case vignette “rape” and criteria according to Briken and Müller (2014): 88 participants; case vignette “abuse” and criteria according to Boetticher et al. (2005): 90 participants; and case vignette “abuse” and criteria according to Briken and Müller (2014): 82 participants.

The study was approved by the ethic committee of Hamburg Chamber of Psychotherapists on the 07.12.2018.

## Results

Table 1 shows the absolute and relative frequencies with regard to the correct assessment of the SASA and diminished capacity in total and with regard to the differentiation between the two sets of criteria. The data show that the evaluators were more successful in correctly predicting the diminished capacity than the SASA.

**TABLE 1:** Absolute and Relative frequencies with regard to the correct assessment of the SASA and diminished capacity.

		SASA		Diminished capacity	
			
Correct assessments		Absolute frequency	Relative frequency	Absolute frequency	Relative frequency
Total		233	66.8%	261	74.8%
Criteria set	Boetticher et al.	105	58.7%	122	68.2%
	Briken and Müller	128	75.3%	139	81.8%

The logistic regression model (Table 2) shows that the chance of correctly assessing the SASA using the Briken and Müller (2014) criteria was 2.15 times higher than using those by Boetticher et al. (2005). With regard to the correct assessment of the diminished capacity, it was shown that the chance for a correct prediction with the criteria catalog by Briken and Müller (2014) was 2.10 times higher than with the criteria catalog according to Boetticher et al. (2005).

**TABLE 2:** Logistic regression model with regard to the different criteria sets.

Model	SASA			Diminished capacity		
		
	Odds ratio	95%-KI	*p*-value	Odds ratio	95% KI	*p*-value
Model 1: Criteria set (Reference: Boetticher et al.)	2.15	1.36–3.40	0.001	2.10	1.27–3.46	0.004

Looking at the interactions between the two case vignettes and the two criteria sets, there were no significant differences: with regard to the SASA, the interaction *p*-value was 0.084 and the diminished capacity was 0.459. As a consequence, the comparison between the two criteria catalogs took place independently of the case vignette—in other words: the two case vignettes behaved similarly.

Table 3 shows the result of the logistic regression model of the items of both criteria sets with regard to the variable SASA corrected for the vignettes. None of the items alone yielded a significant result in the prediction of SASA.

**TABLE 3:** Logistic regression model of the items of both criteria sets with regard to the variable SASA.

Criteria set	Items	Wald-Chi-Quadrat	df	*p*-value
Boetticher et al.	The paraphilic disorder determines the sexuality of the person to a great extent.	0.09	2	0.955
	Paraphilic impulses are denied and perceived as egodystonic (=alien). While being overcontrolled and masked the vast amount of time, the ability to control the impulses decreases significantly under certain circumstances.	5.27	2	0.072
	The paraphilic disorder is progressive with regard to its dynamic, that is, the person being evaluated experiences themselves as progressively being flooded by stronger and stronger paraphilic impulses: The pressure to act on them increases.	2.94	2	0.230
	The person has (as a result of particular personality factors or sexual dysfunction) few or no other opportunities to satisfy himself or herself sexually.	2.32	2	0.313
Briken and Müller	Paraphilic sexual interests.	<0.01	1	0.951
	Sex drive/Sex preoccupation.	4.76	2	0.093
	Sex as Coping.	1.24	2	0.539
	Capacity for stable relationships.	0.30	2	0.862
	General social rejection.	0.58	2	0.749

Table 4 shows the result of the logistic regression model of the items of both criteria catalogs with regard to the variable diminished capacity. The choice of a specific category of the item “Conflictual escalation and emotional weakening before the suspected offense with an already long-standing hopelessness in the dynamic of sexual drive” had a significant influence on the correct assessment of diminished capacity, irrespective of the vignette and the answers to the other items.

**TABLE 4:** Logistic regression model of the items of both criteria sets with regard to the variable diminished capacity.

Criteria set	Items	Wald-Chi-Quadrat	df	*p*-value
Boetticher et al.	Conflictual escalation and emotional weakening before the suspected offense with an already long-standing hopelessness in the dynamic of sexual drive.	9.64	2	0.008
	Carrying out of the offense, even in social situations that are strictly monitored.	0.67	2	0.716
	An abrupt, impulsive course of criminal action.	4.60	2	0.100
	Criminal action that appears ritualized. Outer stimuli appear to be blanked out.	5.65	2	0.059
	Contributing factors (substance intoxication, co-occurring disorders, personality disorders, limited intelligence).	1.80	2	0.406
	Indicators for preparations being made for the offense in advance, as well as a planned approach (elaborations of paraphilic fantasies are expressly not included here).	0.41	2	0.814
	The ability to wait or a crime that is very prolonged in nature.	4.62	2	0.099
	The crime involves a complex step-by-step sequence of actions.	4.09	2	0.130
	Precautions are taken against being discovered.	2.79	2	0.249
	The person has previously acted differently in comparable situations.	3.41	2	0.182
Briken and Müller	Sexual pre-occupations.	0.50	2	0.781
	Emotional collapse.	5.90	2	0.052
	Collapse of social support.	2.20	2	0.332

Table 5 shows the results of the logistic regression model of the item “Conflictual escalation and emotional weakening before the suspected offense with an already long-standing hopelessness in the dynamic of sexual drive” with regard to the variable diminished capacity. The parameter estimates are corrected for the other items and for the vignettes (adjusted model). The chance that diminished capacity was correctly assessed was 9.81 times higher if the item was assessed as “completely present” compared to “not present at all.”

**TABLE 5:** Logistic regression model of the item “Conflictual escalation and emotional weakening before the suspected offense with an already long-standing hopelessness in the dynamic of sexual drive” with regard to the variable diminished capacity.

Item: “Conflictual escalation and emotional weakening before the suspected offense with an already long-standing hopelessness in the dynamic of sexual drive”	Odds ratio	95%-Wald-CI: Lower	Upper
Specification: “is completely present” (Reference specification: “is not present at all”)	9.81	2.21	43.50
Specification: “is completely present” (Reference specification: “is somewhat present”)	4.48	1.39	14.49
Specification: “is somewhat present” (Reference specification: “is not present at all”)	2.18	0.67	7.13

## Discussion

In this study, the predictive validity based on the criteria of Briken and Müller (2014) was higher than the predictive validity of the criteria of Boetticher et al. (2005). This applied both for the assessment with regard to the SASA and for the diminished capacity. Possible reasons for this could be that the items by Briken and Müller (2014), in contrast to the criteria by Boetticher et al. (2005), have been operationalized more in detail, so there should be fewer incongruities in the understanding of the individual items and consequently, the content of the target variables SASA and diminished capacity can be captured better.

The analysis on the item level showed that only the item “Conflictual escalation and emotional weakening before the suspected offense with an already long-standing hopelessness in the dynamic of sexual drive” from the criteria catalog of Boetticher et al. (2005) had a significant influence on the correct prediction of diminished capacity, independent of the vignette and the answers regarding the other items. Maybe, this item seems to play a special role for the decision process.

From a statistical point of view, sufficient reliability applies as a prerequisite for the validity of diagnostic procedures. In addition, for the practical application of diagnostic criteria catalogs, it is advantageous if the measured target variables can be captured with as few items as possible. From a preliminary study by Dobbrunz et al. (2020), we know that the IRR based on the criteria of Briken and Müller (2014) are higher than those of the criteria of Boetticher et al. (2005). To sum up, there are indicators pointing toward the notion that Briken and Müller’s criteria catalog (2014) might represent a more suitable procedure—both in terms for the assessment of the SASA as well as for the diminished capacity. However, the validity of both criteria catalogs should be investigated in further studies using real cases. Results should also be discussed in qualitative studies by experts.

### Implications and Limitations

It should be pointed out that the evaluations were based on two constructed and non-comprehensive case vignettes which were kept relatively short for reasons of time efficiency and to be applicable for use in future studies. Furthermore, the experts lacked a personal impression of the subject, as is usually the case in a psychological/psychiatric assessment or a possible main trial in court. In order to specifically measure the effect of the assessment of paraphilic disorders in this study, the inclusion of other disorders into the case vignettes was omitted. This reduction in complexity surely leads to the fact that the cases presented are less similar to naturalistic cases. In reality, comorbid diagnoses (e.g., with personality disorders) are often present in the context of assessing criminal responsibility in sexual offenders. Varying the cases with further diagnoses would have meant a different methodological approach and an even larger sample. This would have made the implementation of this study more difficult. However, comorbid diagnosis and constellative factors should be considered in future studies.

## Conclusion

It may currently make sense to include both criteria catalogs in the assessment process for greater transparency and to further investigate this approach in research. However, the results so far already indicate that the criteria of Briken and Müller (2014) are superior to those of Boetticher et al. (2005) in terms of predictive validity. This can also be assumed for IRR. However, the proposed criteria should not be seen as the only source of information in the assessment process.

## Data Availability Statement

The raw data supporting the conclusions of this article will be made available by the authors, without undue reservation.

## Author Contributions

SD and PB are the main authors. AD gave statistical expertise. JM assisted in the discussion. All authors contributed to the article and approved the submitted version.

## Conflict of Interest

The authors declare that the research was conducted in the absence of any commercial or financial relationships that could be construed as a potential conflict of interest.

## Appendix

Case vignette 1: The defendant Mr. Z. is 46 years old (^∗^12.01.1972). He is accused of repeatedly sexually abusing 4-year-old Jonas, the son of his partner, over a period of approximately 2 years. On the basis of pictures and video tapes, which were produced during the sexual acts by Mr. Z., 38 single-acts were identified. Mr. Z. performed oral sex on the boy and penetrated him anally, in doing so the boy cried. Mr. Z. was always alone in the shared apartment with Jonas when performing these acts. He payed meticulous attention to this fact. During the questioning of the witnesses, the partner and the sister, it turned out that they regarded the accusations as unimaginable. Moreover, the partner stated that she had always had—during the 2 and a half year relationship with Mr. Z.—the impression that he had little interest in mutual sexual activity, but he had explained this as a results of his workload. In the last months of the relationship, there had been nearly no sexual intercourse. Mr. Z. had lost his job as a building cleaner a few weeks ago. In the examination of the defendant by the criminal investigation department, Mr. Z. stated that he had initially begun to caress the boy and had expanded the intensity of this act gradually in the process. Furthermore Mr. Z. stated that he had recorded the act with his digital camera to be able to watch it again anytime later—for example when feeling stressed or after conflicts with his partner or at work. Mr. Z. had fallen in love for the first time in his schooldays (seventh grade) with a classmate of the same age. In his adult life, he maintained affairs and short relationships that lasted up to 3 years. He had relationships with women and with men which he experienced as mutually satisfying. The age of the male partners had decreased over time. Currently Mr. Z. preferred boys at the age of 4–6 years. He had used homosexual pornography since his youth, however, over time he consumed more and more child pornography. Mr. Z. was in regular contact with his family of origin and had two best friends which he had known since their schooldays. There was no quantitative increase in the sexual assaults to the detriment of Jonas after the termination of his work contract a few weeks ago. He made use of child pornography and masturbated to it (approximately five times a week). The termination of his work contract had lowered his self-esteem temporarily, but he was confident to find a new employment soon. Regarding the delinquent prehistory: At the age of 27 Mr. Z. had been accused of approaching boys aged of 6–12 in a swimming team with a sexual intention; the proceeding was dismissed.

Case vignette 2: The defendant Mr. M. is 24 years old (^∗^05.02.1994). He is accused of grabbing the 48 year old Mrs. N, who was passing by, from the back in the evening hours of the 10.03.2018 around 8 pm on an illuminated sidewalk in front of the front door of his residential house (multi-family house with 12 rental parties). He kept her mouth shut with his left hand, fixated her upper arm with his right hand, and dragged the woman, who tried to defend herself, to his rented apartment, which was situated on the first floor. Arriving in the apartment he violently headbutted the woman, who continuously cried for help, with a hammer so that she lost consciousness. Then he taped the woman’s mouth shut with package tape and tied her wrists and ankles up with cable ties. Afterward he masturbated twice until ejaculation in front of the still unconscious woman. As Mrs. N. gained consciousness, approximately 3 h later, Mr. M. demanded oral sex by grabbing her hair multiple times and punching her on her throat and on her ears violently multiple times. A neighbor, whose attention was caught by the woman’s screams, eventually called the police.

In the defendant’s examination by the criminal investigation department Mr. M. stated, among other things, that he had already been standing in front of his window for several hours searching for women before he had spotted Mrs. N. Mrs. N. was the first woman who was alone. Thereto he manipulated his member. He had pre-fantasized about violently overpowering an unsuspecting woman and had researched sadistic pornography on the internet. He did not consider neighbors, pedestrians, or other potential interferences in his plan—“I had tunnel vision.” The torture and humiliation of the woman in form of punching and hair ripping was connected to a particular sexual pleasure.

Mr. M. stated moreover that he regularly watched pornography and had done so since his 13th year of life. There would be days in which he would search for stimulating content on the internet for hours (up to 5 h)—his preference would be abduction and rape which included the use of physical violence and humiliation. In times of stress and rejection, he would vent his stress by masturbating, as he would feel better for a while afterward. Except for a relationship with a former classmate that lasted 10 months, in which there had been repeated domestic violence, he had no relationship experiences. Mr. M. visited prostitutes regularly.

Except for his older sister, who also visited Mr. M. in custody regularly, he did not have any noteworthy social contacts. He had only met an elderly man from the opposite apartment to play cards a couple of times. As a result of a dispute with his parents, 14 days before the alleged crime, his parents had cut off contact with him. The reason for the dispute was that Mr. M. had discarded the plan to start an apprenticeship. Here, once again, Mr. M experienced rejection.

Regarding the delinquent prehistory: Mr. M. had been released from juvenile prison 4 weeks prior to the alleged crime, where he had been jailed for a custodial sentence of 3 1/2 years because of sexual harassment in two cases and another case of attempted sexual harassment. Mr. M. had rejected therapy because he considered himself mentally healthy.
